# Optimizing Hospital Performance Evaluation in Total Weight Loss Outcomes After Bariatric Surgery: A Retrospective Analysis to Guide Further Improvement in Dutch Hospitals

**DOI:** 10.1007/s11695-024-07195-4

**Published:** 2024-07-09

**Authors:** Floris F. E. Bruinsma, Ronald S. L. Liem, Simon W. Nienhuijs, Jan Willem M. Greve, Perla J. Marang-van de Mheen, on behalf of the Dutch Audit for Treatment of Obesity Research Group, G. J. D. van Acker, J. Apers, S. C. Bruin, S. M. M. de Castro, S. L. Damen, I. F. Faneyte, J. W. M. Greve, G. van ’t Hof, F. H. W. Jonker, R. A. Klaassen, E. A. G. L. Lagae, B. S. Langenhoff, R. S. L. Liem, A. A. P. M. Luijten, S. W. Nienhuijs, R. M. Smeenk, S. J. M. Smeets, W. Vening, M. Takkenberg, E. de Witte

**Affiliations:** 1 Department of Surgery, Maastricht University Medical Centre, NUTRIM School for Nutrition and Translational Research in Metabolism, P. Debyelaan 25, 6229 HX Maastricht, The Netherlands; 2 Scientific Bureau, Dutch Institute for Clinical Auditing, Leiden, The Netherlands; 3 Department of Surgery, Groene Hart Hospital, Gouda, The Netherlands; 4 Nederlandse Obesitas Kliniek, The Hague and Gouda, The Netherlands; 5 Department of Surgery, Catharina Hospital, Eindhoven, The Netherlands; 6 Nederlandse Obesitas Kliniek Zuid, Heerlen, The Netherlands; 7 Safety & Security Science and Centre for Safety in Healthcare, Delft University of Technology, Delft, The Netherlands

**Keywords:** Total weight loss, Hospital performance, Median-based funnel plot, Performance evaluation

## Abstract

**Introduction:**

Bariatric surgery aims for optimal patient outcomes, often evaluated through the percentage total weight loss (%TWL). Quality registries employ funnel plots for outcome comparisons between hospitals. However, funnel plots are traditionally used for dichotomous outcomes, requiring %TWL to be dichotomized, potentially limiting feedback quality. This study evaluates whether a funnel plot around the median %TWL has better discriminatory performance than binary funnel plots for achieving at least 20% and 25% TWL.

**Methods:**

All hospitals performing bariatric surgery were included from the Dutch Audit for Treatment of Obesity. A funnel plot around the median was constructed using 5-year %TWL data. Hospitals positioned above the 95% control limit were colored green and those below red. The same hospitals were plotted in the binary funnel plots for 20% and 25% TWL and colored according to their performance in the funnel plot around the median. We explored the hospital’s procedural mix in relation to %TWL performance as possible explanatory factors.

**Results:**

The median-based funnel plot identified four underperforming and four outperforming hospitals, while only one underperforming and no outperforming hospitals were found with the binary funnel plot for 20% TWL. The 25% TWL binary funnel plot identified two underperforming and three outperforming hospitals. The proportion of sleeve gastrectomies performed per hospital may explain part of these results as it was negatively associated with median %TWL (*β* =  − 0.09, 95% confidence interval [− 0.13 to − 0.04]).

**Conclusion:**

The funnel plot around the median discriminated better between hospitals with significantly worse and better performance than funnel plots for dichotomized %TWL outcomes.

**Graphical Abstract:**

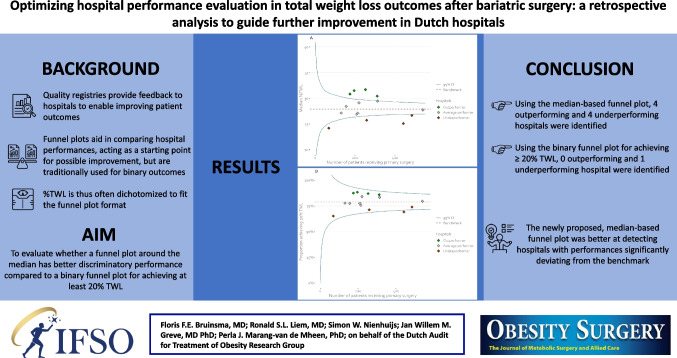

**Supplementary Information:**

The online version contains supplementary material available at 10.1007/s11695-024-07195-4.

## Introduction

Bariatric surgeons aim to achieve the best possible outcomes for their patients, with the percentage of achieved total weight loss (%TWL) [1, 2] used as a primary outcome in many studies. Increasingly, national quality registries are established that provide feedback to healthcare providers on how their performance compares with other providers, which applies to bariatric surgery as well [3, 4]. A frequently used graphical display to give such feedback is the funnel plot. Funnel plots are constructions of control limits around a benchmark which enables identification of hospitals with significantly worse and better outcomes (so called outliers) [5]. The intention in providing this feedback and identifying outlier hospitals is that those with significantly worse performance will investigate the reasons for this performance and then start initiatives to improve their care, which will ultimately benefit patients. As such, funnel plots are useful tools that can give direction for improvement [5–10].

However, funnel plots were designed for binary outcomes, such as mortality or the occurrence of a complication [9, 11, 12] but are now also used for continuous outcomes such as %TWL, which are therefore dichotomized to fit the funnel plot format [13]. For instance, cutoffs are based on a norm or guideline stating whether an outcome is considered good or appropriate. In bariatric surgery, %TWL is often categorized into achieving at least 20% TWL to indicate adequate weight loss [14–16], although in some instances, 25% is regarded as a more favorable indicator for successful treatment [17], and therefore, such dichotomized outcomes are used for comparing hospital performances [13]. However, when dichotomizing continuous outcomes like %TWL rather than using the whole distribution, information gets lost, and thereby also the power to detect differences in performance between hospitals [7, 9]. The use of binary funnel plots in examining hospital performance on %TWL thereby only investigates the tail of the distribution, i.e., whether a hospital has fewer patients achieving 20% TWL, but this does not necessarily mean that patients in that specific hospital in general experience lower %TWL. Using a funnel plot that would show whether the entire distribution of %TWL is significantly different from other providers might therefore point to additional possibilities for improvement.

Hence, the aim of the current study was to compare hospitals identified as outlier based on a funnel plot around the median %TWL at 5 years versus binary funnel plots of achieving at least 20% and 25% TWL. Furthermore, the current study will explore possible reasons for the performance of outlier hospitals.

## Methods

### Setting

The data used for this study were derived from the Dutch Audit for Treatment of Obesity (DATO). DATO is a nationwide, mandatory quality registry for metabolic and bariatric surgery in the Netherlands that collects data on patient characteristics, procedures, complications, and follow-up since 2015 [18, 19]. On-site data verification has proven high validity of the data [20]. All Dutch bariatric clinics participate in this registry, thereby gaining valuable insights in the quality of bariatric care in everyday clinical practice. Healthcare quality is monitored through indicators that provide national benchmarks including the percentage of patients achieving at least 20% TWL during follow-up from 1 up to 5 years after surgery.

The study protocol was approved by the DATO scientific committee. In accordance with Dutch regulations informed consent was not obtained, as DATO is an opt-out registry. The current study was performed in accordance with the ethical standards as stated in the declaration of Helsinki of 1964 and its later amendments.

### Patients, Definitions, and Outcomes

Weight loss expressed in %TWL at 5 years was the basis for the primary analysis, in line with the objective to achieve the best long-term outcomes. The outcome %TWL is calculated as [weight at screening – weight at follow-up] / weight at screening × 100%. All patients who underwent primary bariatric surgery between October 1, 2016, and September 30, 2017, with registered weight at baseline and at 5 years were considered eligible for analysis, which resulted in 15 hospitals being analyzed. Follow-up years are defined in DATO with an approximation of + / − 3 months, meaning that any follow-up between 9 and 15 months is considered a 1-year follow-up moment, and any follow-up between 57 and 63 months is considered a 5-year follow-up moment, thereby taking follow-up until January 1, 2023, into account. As national policies and regulations do not permit patient-linkage between hospitals, potential revisional surgery after the primary surgery could not be accounted for.

Hospital performance and outlier status were compared between a funnel plot around the median %TWL, and binary funnel plots using two different cutoff points, i.e., achieving at least 20% and 25% TWL. The cutoff 20% is commonly used with 25% added from a perspective of continuous quality improvement, as done in previous studies [13, 17, 21]. Outlier status means performing either significantly better (outperformer) or worse (underperformer) than the national benchmark.

### Statistical Analysis

First, the %TWL distribution at 5 years was analyzed by plotting a histogram, which was also used to determine the nationwide median and percentage of patients achieving 20% TWL and 25% TWL. Histograms were also created for each hospital separately, to explore possible differences in distributions. Second, a funnel plot around the median was created which compared the median %TWL of each hospital to the nationwide median, with outliers given a color according to their position with respect to the 95% control limits. Hospitals positioned below the 95% control limit (underperformers) were colored red, hospitals above the 95% control limit (outperformers) were colored green, and hospitals within the control limits were performing conform the nationwide median and therefore colored grey (see appendix for statistical code to create the funnel plot around the median). The median rather than the mean %TWL per hospital was chosen because of its better representation of the overall distribution. Third, the binary funnel plot for achieving at least 20% TWL (yes/no) was created, and hospitals were depicted in this funnel plot using the colors reflecting their performance from the funnel plot around the median as described above. In this way, it is shown how hospitals with a significantly worse (i.e., lower) %TWL distribution would have been missed, i.e., considered performing conform the nationwide benchmark in the binary funnel plot, and thereby missed the incentive to investigate and start improvement initiatives. Fourth, the same analyses were repeated with the binary funnel plot for achieving at least 25% TWL (yes/no).

### Post-Hoc Exploratory Analysis

Decisions on procedure type may explain differences in the %TWL distribution, which may be based on hospital preference rather than patient-mix, as shown in a previous study [13]. Sleeve gastrectomy (SG) and gastric bypass procedures (i.e., Roux-en-Y gastric bypass (RYGB), banded RYGB, or one anastomosis gastric bypass (OAGB), depending on the hospital’s preference), are the two types of surgery that are practiced most. Therefore, the proportion of these procedures performed per hospital was included as the independent variable in a linear regression analysis for the outcome %TWL. As RYGB is the most commonly performed surgery in the Netherlands [4], the proportion of this type of gastric bypass was analyzed separately. This approach will provide insight whether a difference in %TWL distribution may be driven by the choice in procedure type, which could be among the things for underperforming hospitals to investigate. In case of an identified association, funnel plots were separately constructed for SG and RYGB as well to explore whether hospital variation remains within patients undergoing these procedures.

### Sensitivity Analysis

As feedback with funnel plots supports local improvement cycles, it could be preferable to have feedback on outcomes that are achieved by more recent treatment strategies, such as 1-year outcomes. Therefore, similar funnel plots as in the primary analysis were constructed using the outcome %TWL at 1 year for patients operated in the same period (i.e., October 1, 2016, until September 30, 2017). In this way, it was possible to examine whether the same hospitals are identified as outliers in the funnel plot for 1- and 5-year outcomes, thereby exploring whether the performance at 1 year is predictive for their performance at 5 years. The same approach as in the primary analysis was used for analyzing choice of procedure type as an explanatory factor.

### Validation

To validate the performance of the median-based funnel plot in a different patient cohort, we created funnel plots for the outcome %TWL at 1 year including all patients receiving primary surgery in 2021 (i.e.,operated between October 1, 2020, and September 30, 2021). All statistical analyses were performed using RStudio version 2023.06.1 (R Foundation for Statistical Computing, Vienna, Austria).

## Results

**Fig. 1:**
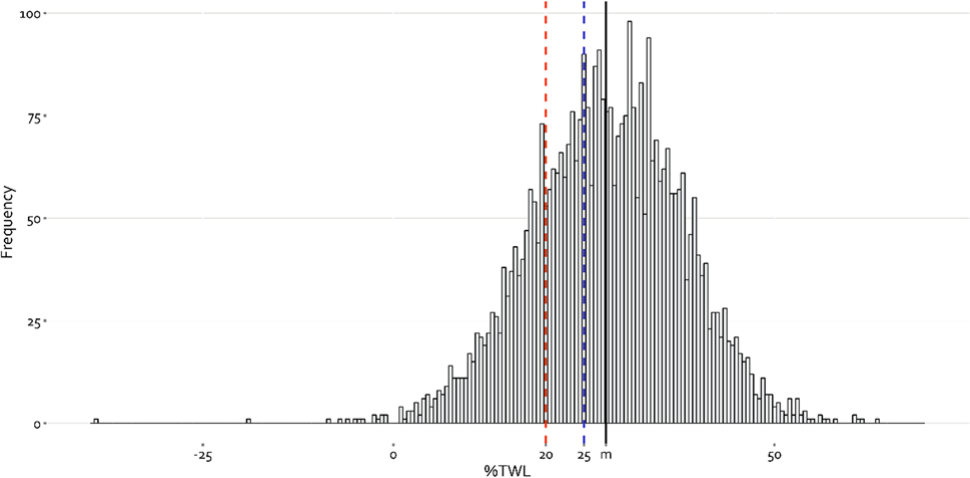
Histogram of %TWL outcomes at 5 years showing an approximately normal distribution. Left from the dashed orange line are all patients with < 20% TWL and left from the dashed blue line are all patients with < 25% TWL. The solid black line displays the position of the median (m)

Between October 1, 2016, and September 30, 2017, 8907 patients received bariatric surgery. Of these, 3971 patients (44.6%) had registered follow-up weight at 5 years and were therefore included in the analysis. As shown in Fig. 1, %TWL at 5 years followed a normal distribution with the median TWL at 27.9%, and overall, 78.8% of patients achieved ≥ 20% TWL and 62.4% achieved ≥ 25% TWL. Normal %TWL distributions were found for all hospitals (see supplementary Fig. 1).

**Fig. 2:**
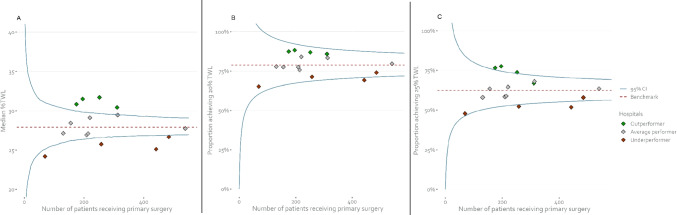
Total weight loss outcomes at 5 years per hospital, displayed in three different types of funnel plots. Each diamond represents a hospital. **A** Funnel plot constructed around the nationwide median %TWL. The median %TWL of hospitals falling above the 95% control limit was significantly higher than the nationwide median and these are therefore colored green. Hospitals falling below the 95% control limit performed significantly worse than the nationwide median and are therefore colored red. **B** Funnel plot constructed for the binary outcome ≥ 20% TWL (yes/no). Hospitals are colored according to their performance in the funnel plot around the median. **C** Funnel plot constructed for the binary outcome ≥ 25% TWL (yes/no). Hospitals are colored according to their performance in the funnel plot around the median. Average performer means that the hospital performed consistent with the nationwide median. TWL = total weight loss, CI = confidence interval

As shown in Fig. 2A, four hospitals had significantly better distribution of 5-year %TWL than the nationwide median and were therefore depicted in green. In addition, four hospitals had significantly worse distribution, i.e., lower %TWL than the nationwide median and were therefore depicted in red. Figure 2B shows that these hospitals with significantly better %TWL distribution would not have been identified with a binary funnel plot using the 20% TWL cutoff, and that only one of the underperforming hospitals with significantly worse %TWL distribution would have been identified. By not getting a signal of underperformance, these hospitals would likely not have initiated any improvement initiatives to improve weight loss.

Figure 2C shows that using the 25% TWL threshold in a binary funnel plot would have identified three of the four outperforming hospitals and two of the four underperforming hospitals, so also with this higher threshold there would be hospitals not getting a signal when a binary funnel plot was used even though they overall achieved less favorable %TWL results. Hospitals with a performance consistent with the nationwide median were never an outlier on the binary funnel plots.

### Post-Hoc Exploratory Analysis

In the exploratory analysis, a higher proportion of SG performed per hospital was associated with lower median %TWL at 5 years (*β* =  − 0.09; 95% CI [− 0.13 – − 0.04], *P* < 0.001), which translates to a decline of 0.9% TWL for every 10%-points increase in proportion of SG performed. On the other hand, the proportion of gastric bypass performed (i.e., RYGB, banded RYGB, or OAGB) was associated with higher median %TWL (*β* = 0.09; 95% CI [0.04–0.13], *P* < 0.001), while the proportion of solely RYGB was not associated with %TWL at 5 years (*β* =  − 0.01, 95% CI [− 0.06, 0.04], *P* = 0.71). Furthermore, stratified by procedure type (i.e., SG and RYGB), variation between hospitals remained as shown in the funnel plots around the median in supplementary figs. 2 and 3, indicating that non-procedure-related aspects influence the performance on the %TWL distribution as well.

### Sensitivity Analysis

**Fig. 3:**
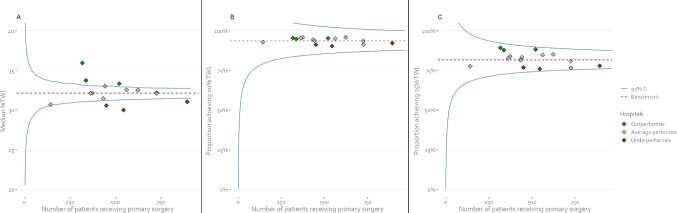
Total weight loss outcomes at 1 year per hospital, displayed in three different types of funnel plots. Each diamond represents a hospital. **A** Funnel plot constructed around the nationwide median %TWL. The median %TWL of hospitals falling above the 95% control limit was significantly higher than the nationwide median and these are therefore colored green. Hospitals falling below the 95% control limit performed significantly worse than the nationwide median and are therefore colored red. **B** Funnel plot constructed for the binary outcome ≥ 20% TWL (yes/no). Hospitals are colored according to their performance in the funnel plot around the median. **C** Funnel plot constructed for the binary outcome ≥ 25% TWL (yes/no). Hospitals are colored according to their performance in the funnel plot around the median. Average performer means that the hospital performed consistent with the nationwide median. TWL = total weight loss

Of the patients operated between October 1, 2016, and September 30, 2017, 7,461 cases (83.8%) had registered weight at 1 year and were therefore included in the sensitivity analysis. Median TWL at 1 year was 32.1%, with 93.7% and 81.7% of patients achieving ≥ 20% TWL and ≥ 25% TWL, respectively. Figure 3 shows the differences in identification of better and worse performers in a funnel plot around the nationwide median and for achieving ≥ 20% and ≥ 25% TWL, with no outliers being detected in both binary funnel plots, while three underperformers and three outperformers were identified in the funnel plot around the median. No significant association with %TWL at 1 year was found for the proportion of performed SG (*β* =  − 0.03, 95%CI [− 0.07–0.00], *P* = 0.052), gastric bypass (*β* = 0.03, 95%CI [0.00–0.07], *P* = 0.053), or RYGB (*β* =  − 0.02, 95% CI [− 0.05, 0.01], *P* = 0.21).

Two of the three underperforming hospitals at 1 year were also underperforming hospitals at 5 years and all three outperforming hospitals at 1 year were also outperformers at 5 years, as shown in Table 1.

**Table 1:**
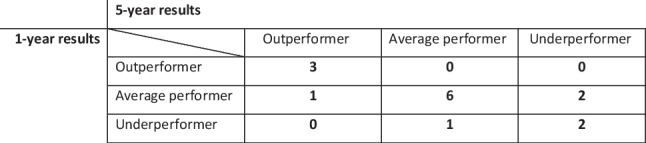
Concordance of the 1-year performance with the 5-year performance according to the position in the funnel plots around the median for the outcomes %TWL at 1 and 5 years. Average performer means that the hospital performed consistent with the nationwide median

### Validation

When analyzing patients from a different cohort (i.e., operated between October 1, 2020, and September 30, 2021), the analysis yielded similar results. As shown in supplementary Fig. 4, the median-based funnel plot identified 9 hospitals with outlier performance (4 underperformers and 5 outperformers) on %TWL outcomes at 1 year, with none of these hospitals identified when using the binary funnel plots.

## Discussion

The current study showed that for the continuous outcome %TWL at 5 years, a funnel plot around the median had better discrimination compared to funnel plots for the dichotomized outcomes ≥ 20 and ≥ 25%TWL. Four hospitals were identified as achieving a significantly better %TWL distribution at 5 years, which would have been missed when a binary funnel plot with a 20% cutoff was used, and only three of these were identified using the 25% cutoff. Four hospitals were identified as achieving significantly worse %TWL distribution, with only one of these identified when using the 20% cutoff, and two when the 25% cutoff was used. The exploratory analysis showed that a higher proportion of SGs performed was associated with achieving lower %TWL at 5 years, suggesting that choice of procedure partly explains why some hospitals achieve overall worse %TWL results although part of the variation between hospitals remained even when stratified by procedure.

The use of funnel plots for evaluating hospital performance is not new [3, 5, 10, 22, 23], but continuous outcomes are often dichotomized to fit a binary funnel plot format rather than that a funnel plot for a continuous outcome is used. [13, 24] The current study showed that use of a binary funnel plot for continuous outcomes resulted in suboptimal feedback, as fewer hospitals were identified by the commonly used cutoffs for achieving adequate %TWL, thereby showing the added value of this new type of funnel plot. Furthermore, since all outliers in the binary funnel plot were also outliers in the funnel plot around the median, the binary funnel plot appears to have no advantages. This might be due to %TWL being normally distributed, but could be different for a skewed distribution (e.g. many patients not achieving 20%TWL despite the hospital’s median not deviating from the nationwide median) [25]. For such skewed distributions, both types of funnel plots can be used together, as they highlight whether improvement should be pursued for all patients or only for a subpopulation not achieving the specific threshold [25]. However, for the normally distributed outcome %TWL, there appears to be no added value of using a binary funnel plot.

The association between the proportion of SGs performed per hospital and lower %TWL at 5 years found in the post hoc exploratory analysis, is in line with findings of previous studies [26–28]. In contrast, such an association was not found for %TWL at 1 year. The disparity in these findings may be attributable to the increased occurrence of weight recurrence after 1 year among patients receiving SG, as shown in a previous study [29]. Consequently, the two hospitals with the highest SG percentages showed a performance consistent with the national median regarding %TWL at 1 year but were underperformers at 5 years. Notably, the proportion of RYGB procedures performed per hospital was not associated with %TWL, suggesting that other gastric bypass procedures than RYGB might result in the highest %TWL, such as OAGB or banded gastric bypass techniques [30, 31]. In addition, since variation in performance persisted when the funnel plots were stratified by procedure type, non-procedure-related factors likely influence %TWL as well. The degree of achieved preoperative weight loss or patient characteristics such as socio-economic status could explain part of the remaining variation [32, 33], but additional research might reveal further factors responsible for achieving a significantly better %TWL distribution.

The sensitivity analysis for %TWL at 1 year showed the superior performance of the funnel plot around the median as well, revealing three underperforming and three outperforming hospitals. Both binary funnel plots were not able to identify any outliers, probably because the threshold values 20% and 25% were too distant from the national median of 32.1% TWL, with only 6.3 and 18.3% of patients falling below these thresholds, respectively. When comparing the funnel plot around the median for the 1- and 5-year outcomes, a hospital’s position in the funnel plot for the 1-year outcome predicted their position in the funnel plot for the 5-year outcomes in most cases. This suggests that short-term results are indicative for the hospital’s long-term weight loss outcomes.

In current practice, many quality indicators are based on dichotomous variables or are dichotomized using cutoffs as done for %TWL [13, 18]. For continuous variables, the current study showed that national quality registries should likely replace the binary funnel plot for a more suitable funnel plot that incorporates information from the entire distribution such as the funnel plot around the median, or at least add the latter funnel plot depending on the variable’s distribution. Because the funnel plots around the median were better able to discriminate between healthcare providers in their performance, they can provide hospitals with an incentive to search for explanations for the performance. To date, Dutch hospitals were assumed to all have similar weight loss results because of the way the results were presented to them. If a switch were to be realized, the motivation for hospitals to further optimize weight loss results could be invigorated. Although the achieved weight loss can be considered satisfactory and good for all hospitals, in the context of continuous quality improvement one should continue to strive for the best possible patient outcomes. The current study shows how some hospitals may still improve further. As the 1-year performance was predictive in many cases for long-term outcomes, the funnel plot around the median could assist in the early discovery of suboptimal weight loss, which could result in initiatives to reconsider local protocols or treatment strategies.

Some limitations of the current study should be noted. First, it must be considered that good clinical performance entails more than just weight loss. Other outcomes are equally important, such as improvement of comorbidities, absence of complications, and patient-reported outcomes. Therefore, it should be noted that attaining the greatest weight loss does not necessarily reflect the best outcome for the patient. Subsequently, in the future, other outcomes should be created to evaluate outcomes of bariatric surgery and complement the %TWL funnel plot, for example a composite outcome measure including all the aforementioned aspects involving optimal outcome. Nevertheless, to date, Dutch hospitals were thought to have similar weight loss outcomes, as the binary funnel plot was mostly not able to show variation, whereas in fact the new funnel plot pointed out that differences do exist. Together with other quality indicators such as complication rates [12, 18], it thereby enables better self-evaluation for bariatric clinics. Second, the current study did not consider whether patients received revisional surgery, such as conversion to another technique, which could have influenced %TWL. However, it is likely that patients receiving SG more often experienced weight recurrence with subsequent revisional surgery, and therefore these patients would have experienced even lower %TWL if revisional surgery had not been performed. [29] Therefore, the assumption that the proportion of SGs performed is associated with lower %TWL at 5 years can still be deemed valid. Last, future research should reveal whether applying the median-based funnel plot in other populations yields similar results. The current study showed that the superiority of the median-based funnel plot persisted following (internal) validation in a different patient cohort. However, since the distribution of %TWL might be different in other countries, further validation in a different country would confirm its added value.

## Conclusion

In the pursuit of improving healthcare-related outcomes, discovering variation between hospitals is an important first step. Funnel plots are then a useful tool, but the way in which such a funnel plot is constructed is of great importance. When variation is found, the next step is to search for explanations for this variation before improvement initiatives can be initiated. The current study elucidated that for %TWL, a funnel plot around the median had better discrimination than a funnel plot for the dichotomized outcomes ≥ 20 and ≥ 25% and therefore should preferably be used when comparing weight loss outcomes so that the entire distribution is taken into account.

## Supplementary material

Below is the link to the electronic supplementary material.

Supplementary file1 (DOCX 178 KB)

Supplementary file2 (DOCX 149 KB)

Supplementary file3 (DOCX 148 KB)

Supplementary file4 (DOCX 232 KB)

Supplementary file5 (DOCX 160 KB)

## References

[1] van Rijswijk AS, van Olst N, Schats W, et al. What is weight loss after bariatric surgery expressed in percentage total weight loss (%TWL)? A systematic review. Obes Surg. 2021;31(8):3833–47. 34002289 10.1007/s11695-021-05394-x

[2] Van De Laar AW, Emous M, Hazebroek EJ, et al. Reporting weight loss 2021: position statement of the Dutch Society for Metabolic and Bariatric Surgery (DSMBS). Obes Surg. 2021;31(10):4607–4611. 10.1007/s11695-021-05580-x 34283377

[3] Beck N, Van Bommel AC, Eddes EH, et al. The Dutch Institute for clinical auditing: achieving Codman’s dream on a nationwide basis. Ann Surg. 2020;271(4):627–31. 31972639 10.1097/SLA.0000000000003665

[4] Brown W, Kow L, Anvari M, et al. IFSO - 8th Global Registry Report. 2023;1–76.

[5] Spiegelhalter DJ. Funnel plots for comparing institutional performance. Stat Med. 2005;24(8):1185–202. 15568194 10.1002/sim.1970

[6] van der Willik EM, van Zwet EW, Hoekstra T, et al. Funnel plots of patient-reported outcomes to evaluate health-care quality: basic principles, pitfalls and considerations. Nephrology (Carlton). 2021;26(2):95. 32725679 10.1111/nep.13761 PMC7891340

[7] Seaton SE, Manktelow BN. The probability of being identified as an outlier with commonly used funnel plot control limits for the standardised mortality ratio. BMC Med Res Methodol. 2012;12:98. 22800471 10.1186/1471-2288-12-98 PMC3441904

[8] Verburg IWM, Holman R, Peek N, et al. Guidelines on constructing funnel plots for quality indicators: a case study on mortality in intensive care unit patients. Stat Methods Med Res. 2018;27(11):3350. 28330409 10.1177/0962280217700169 PMC6193208

[9] Seaton SE, Barker L, Lingsma HF, et al. What is the probability of detecting poorly performing hospitals using funnel plots? BMJ Qual Saf. 2013;22(10):870–6. 23832924 10.1136/bmjqs-2012-001689

[10] Gale CP, Roberts AP, Batin PD, et al. Funnel plots, performance variation and the Myocardial Infarction National Audit Project 2003–2004. BMC Cardiovasc Disord. 2006;2:6. 10.1186/1471-2261-6-34 PMC1555633 16884535

[11] Elfrink AKE, van Zwet EW, Swijnenburg RJ, et al. Case-mix adjustment to compare nationwide hospital performances after resection of colorectal liver metastases. Eur J Surg Oncol. 2021;47(3):649–59. 33183927 10.1016/j.ejso.2020.10.016

[12] Poelemeijer YQM, Marang-van de Mheen PJ, Wouters MWJM, et al. Textbook Outcome: an Ordered Composite Measure for Quality of Bariatric Surgery. Obes Surg. 2019 Apr;29(4):1287–1294. 10.1007/s11695-018-03642-1 30569369

[13] Akpinar EO, Liem RSL, Nienhuijs SW, et al. Hospital variation in preference for a specific bariatric procedure and the association with weight loss performance: a nationwide analysis. Obes Surg. 2022;32(11):3589–99. 36100807 10.1007/s11695-022-06212-8 PMC9613549

[14] Barthold D, Brouwer E, Barton LJ, et al. Minimum threshold of bariatric surgical weight loss for initial diabetes remission. Diabetes Care. 2022;45(1):92–9. 34518376 10.2337/dc21-0714 PMC8753771

[15] de Vries L, Van den Broecke C, Decruyeneare A, et al. A SIMPLE performance assessment of bariatric procedures and post-operative weight regain. J Gastrointest Surg. 2022;26(3):542–9. 34668160 10.1007/s11605-021-05172-1

[16] Grover BT, Morell MC, Kothari SN, et al. Defining weight loss after bariatric surgery: a call for standardization. Obes Surg. 2019;29(11):3493–9. 31256357 10.1007/s11695-019-04022-z

[17] Tu Y, Pan Y, Han J, et al. A total weight loss of 25% shows better predictivity in evaluating the efficiency of bariatric surgery. Int J Obes (Lond). 2021;45(2):396–403. 32981929 10.1038/s41366-020-00690-5

[18] Poelemeijer YQM, Liem RSL, Nienhuijs SW. A Dutch nationwide bariatric quality registry: DATO. Obes Surg. 2018;28(6):1602–10. 29273926 10.1007/s11695-017-3062-2 PMC5973991

[19] Beck N, Van Bommel AC, Eddes EH, et al. The Dutch Institute for clinical auditing: achieving Codman’s dream on a nationwide basis. Ann Surg. 2020;271(4):627–31. 31972639 10.1097/SLA.0000000000003665

[20] van der Werf LR, Voeten SC, van Loe CMM, et al. Data verification of nationwide clinical quality registries. BJS Open. 2019;3(6):857. 31832593 10.1002/bjs5.50209 PMC6887678

[21] van de Laar AW, van Rijswijk AS, Kakar H, et al. Sensitivity and specificity of 50% excess weight loss (50%EWL) and twelve other bariatric criteria for weight loss success. Obes Surg. 2018;28(8):2297–304. 29484610 10.1007/s11695-018-3173-4

[22] Van Dishoeck AM, Looman CWN, Van Der Wilden-van Lier ECM, et al. Displaying random variation in comparing hospital performance. BMJ Qual Saf. 2011;20(8):651–7. 21228432 10.1136/bmjqs.2009.035881

[23] Rakow T, Wright RJ, Spiegelhalter DJ, et al. The pros and cons of funnel plots as an aid to risk communication and patient decision making. Br J Psychol. 2015;106(2):327–48. 25123852 10.1111/bjop.12081

[24] Voeten DM, van der Werf LR, van Sandick JW, et al. Length of hospital stay after uncomplicated esophagectomy. Hospital variation shows room for nationwide improvement. Surg Endosc. 2021;35(11):6344–57. 33104919 10.1007/s00464-020-08103-4 PMC8523439

[25] Kuhrij L, Van Zwet E, Van Den Berg-Vos R, et al. Enhancing feedback on performance measures: the difference in outlier detection using a binary versus continuous outcome funnel plot and implications for quality improvement. BMJ Qual Saf. 2021;30(1):38. 32034014 10.1136/bmjqs-2019-009929 PMC7788228

[26] Salminen P, Helmio M, Ovaska J, et al. Effect of laparoscopic sleeve gastrectomy vs laparoscopic Roux-en-Y gastric bypass onweight loss at 5 years among patients with morbid obesity the SLEEVEPASS randomized clinical trial. JAMA - J Am Med Assoc. 2018;319(3):241–54. 10.1001/jama.2017.20313 PMC5833550 29340676

[27] Wölnerhanssen BK, Peterli R, Hurme S, et al. Laparoscopic Roux-en-Y gastric bypass versus laparoscopic sleeve gastrectomy: 5-year outcomes of merged data from two randomized clinical trials (SLEEVEPASS and SM-BOSS). Br J Surg. 2021;108(1):49–57. 33640917 10.1093/bjs/znaa011

[28] Akpinar EO, Liem RSL, Nienhuijs SW, et al. Metabolic effects of bariatric surgery on patients with type 2 diabetes: a population-based study. Surg Obes Relat Dis. 2021;17(7):1349–58. 33762128 10.1016/j.soard.2021.02.014

[29] Akpinar EO, Liem RSL, Nienhuijs SW, et al. Weight recurrence after sleeve gastrectomy versus Roux-en-Y gastric bypass: a propensity score matched nationwide analysis. Surg Endosc. 2023;37(6):4351–9. 36745232 10.1007/s00464-022-09785-8 PMC10234854

[30] Magouliotis DE, Tasiopoulou VS, Tzovaras G. One anastomosis gastric bypass versus Roux-en-Y gastric bypass for morbid obesity: an updated meta-analysis. Obes Surg. 2019;29(9):2721–30. 31172454 10.1007/s11695-019-04005-0

[31] Jense MTF, Palm-Meinders IH, Sigterman-Nelissen R, et al. The benefits of banded over non-banded Roux-en-Y gastric bypass in patients with morbid obesity: a multi-center study. Obes Surg. 2022;32(6):1856. 35366739 10.1007/s11695-022-06024-w PMC9072269

[32] Lodewijks Y, Akpinar E, van Montfort G, et al. Impact of preoperative weight loss on postoperative weight loss revealed from a large nationwide quality registry. Obes Surg. 2022;32(1):26–32. 34713382 10.1007/s11695-021-05760-9

[33] Stenberg E, Näslund I, Persson C, et al. The association between socioeconomic factors and weight loss 5 years after gastric bypass surgery. Int J Obes (Lond). 2020;44(11):2279. 32651450 10.1038/s41366-020-0637-0 PMC7577856

