# Correction: Optimizing Hospital Performance Evaluation in Total Weight Loss Outcomes After Bariatric Surgery: A Retrospective Analysis to Guide Further Improvement in Dutch Hospitals

**DOI:** 10.1007/s11695-024-07542-5

**Published:** 2024-10-10

**Authors:** Floris F. E. Bruinsma, Ronald S. L. Liem, Simon W. Nienhuijs, Jan Willem M. Greve, Perla J. Marang-van de Mheen, on behalf of the Dutch Audit for Treatment of Obesity Research Group

**Affiliations:** 1 Department of Surgery, Maastricht University Medical Centre, NUTRIM School for Nutrition and Translational Research in Metabolism, P. Debyelaan 25, 6229 HX Maastricht, The Netherlands; 2 Scientific Bureau, Dutch Institute for Clinical Auditing, Leiden, The Netherlands; 3 Department of Surgery, Groene Hart Hospital, Gouda, The Netherlands; 4 Nederlandse Obesitas Kliniek, The Hague and Gouda, The Netherlands; 5 Department of Surgery, Catharina Hospital, Eindhoven, The Netherlands; 6 Nederlandse Obesitas Kliniek Zuid, Heerlen, The Netherlands; 7 Safety & Security Science and Centre for Safety in Healthcare, Delft University of Technology, Delft, The Netherlands

**Correction: Obesity Surgery (2024) 34:2820–2827**

10.1007/s11695-024-07195-4

The name of author Perla J. Marang-van de Mheen was tagged incorrectly. “Perla J.” should be tagged as the given name and “Marang-van de Mheen” should be tagged as the family name.

