# Supplementary material for: Optimizing Hospital Performance Evaluation in Total Weight Loss Outcomes After Bariatric Surgery: A Retrospective Analysis to Guide Further Improvement in Dutch Hospitals
Source: Obes Surg. 2024 Jul 9;34(8):2820–7. doi: 10.1007/s11695-024-07195-4 (PMC11289147; doi:10.1007/s11695-024-07195-4)
Supplement: Supplementary file 1 — Supplementary file1 (DOCX 178 KB) [file 11695_2024_7195_MOESM1_ESM.docx]

**Supplementary figure 1**. Distribution of %TWL at 5 years stratified per hospital.


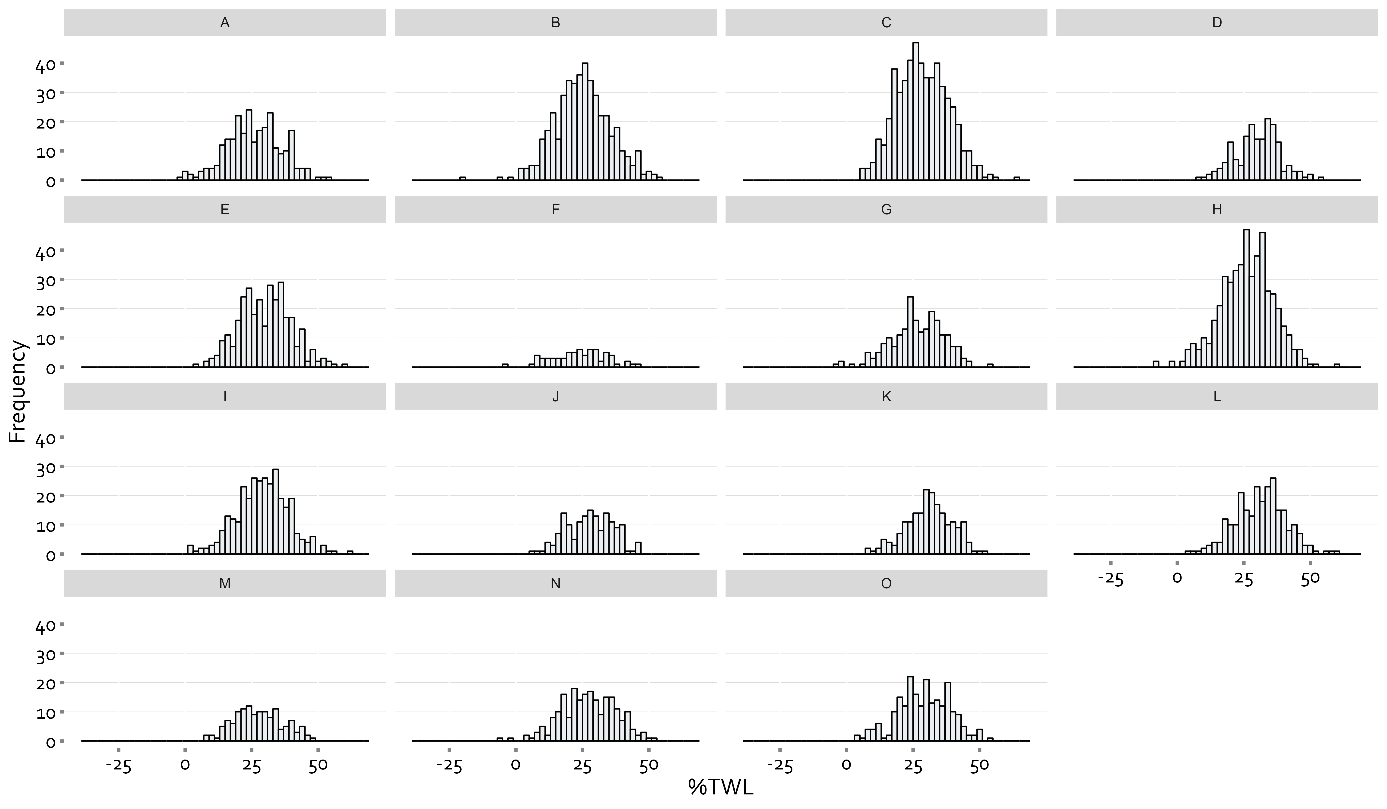


All letters represent a different hospital. TWL = total weight loss
