# Supplementary material for: Optimizing Hospital Performance Evaluation in Total Weight Loss Outcomes After Bariatric Surgery: A Retrospective Analysis to Guide Further Improvement in Dutch Hospitals
Source: Obes Surg. 2024 Jul 9;34(8):2820–7. doi: 10.1007/s11695-024-07195-4 (PMC11289147; doi:10.1007/s11695-024-07195-4)
Supplement: Supplementary file 2 — Supplementary file2 (DOCX 149 KB) [file 11695_2024_7195_MOESM2_ESM.docx]

**
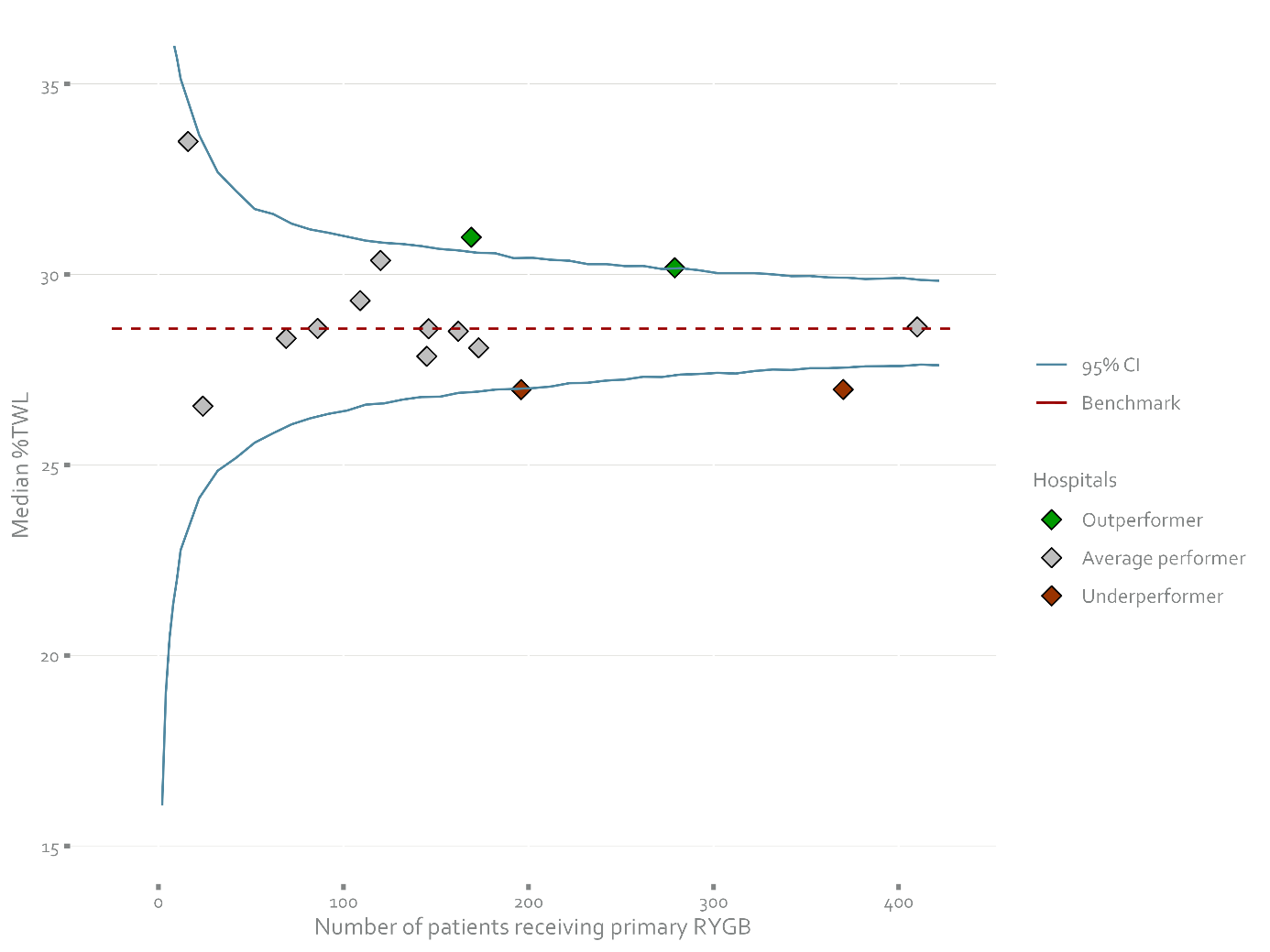
**

**Supplementary figure 2.** Total weight loss outcomes at 5 years per hospital for RYGB procedures. Each diamond represents a hospital. The funnel plot is constructed around the nationwide median %TWL for all patients who received primary RYGB between October 1, 2016, and September 30, 2017. The median %TWL of hospitals falling above the 95% control limit was significantly higher than the nationwide median and these are therefore colored green. Hospitals falling below the 95% control limit performed significantly worse than the nationwide median and are therefore colored red. Average performer means that the hospital performed consistent with the nationwide median. TWL = total weight loss, CI = confidence interval, RYGB = Roux-en-Y gastric bypass.
