# Supplementary material for: Optimizing Hospital Performance Evaluation in Total Weight Loss Outcomes After Bariatric Surgery: A Retrospective Analysis to Guide Further Improvement in Dutch Hospitals
Source: Obes Surg. 2024 Jul 9;34(8):2820–7. doi: 10.1007/s11695-024-07195-4 (PMC11289147; doi:10.1007/s11695-024-07195-4)
Supplement: Supplementary file 4 — Supplementary file4 (DOCX 232 KB) [file 11695_2024_7195_MOESM4_ESM.docx]

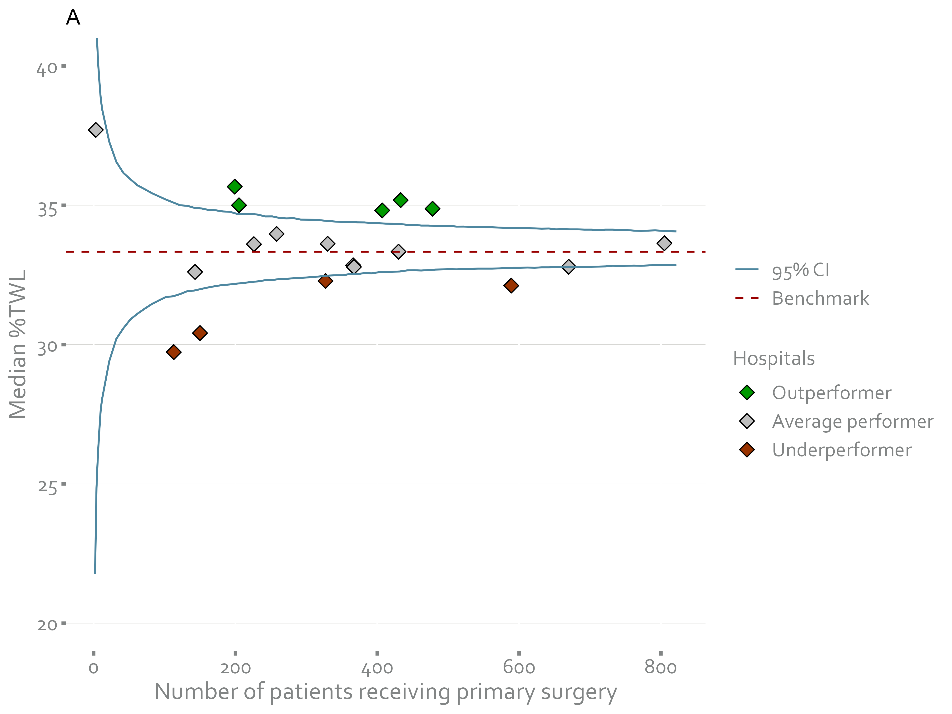

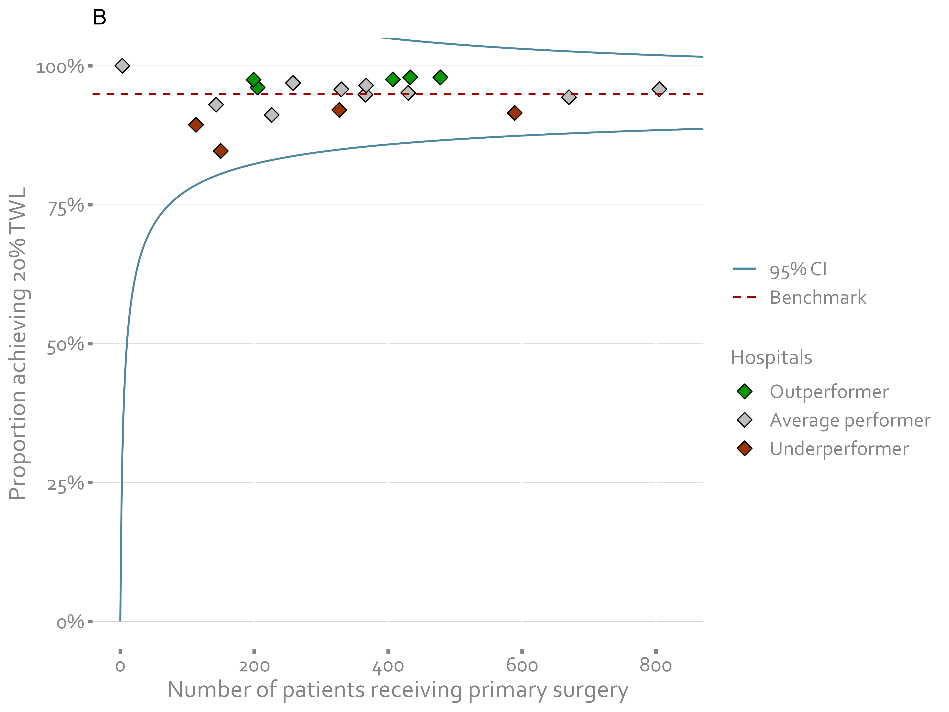

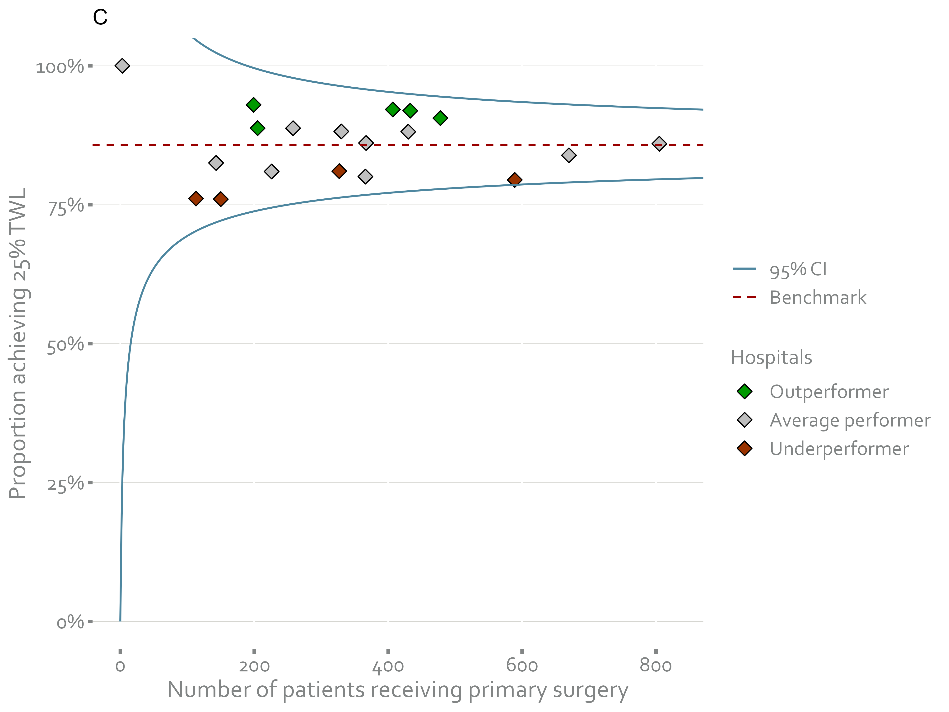


**Supplementary figure 4**. Total weight loss outcomes at 1 year per hospital for patients in the validation cohort (i.e. operated between October 1, 2020, and September 30, 2021) displayed in three different types of funnel plots. Each diamond represents a hospital.

A: Funnel plot constructed around the nationwide median %TWL. The median %TWL of hospitals falling above the 95% control limit was significantly higher than the nationwide median and these are therefore colored green. Hospitals falling below the 95% control limit performed significantly worse than the nationwide median and are therefore colored red. B: Funnel plot constructed for the binary outcome ≥ 20% TWL (yes/no). Hospitals are colored according to their performance in the funnel plot around the median. C: Funnel plot constructed for the binary outcome ≥ 25% TWL (yes/no). Hospitals are colored according to their performance in the funnel plot around the median. Average performer means that the hospital performed consistent with the nationwide median. TWL = total weight loss, CI = confidence interval
