# Supplementary material for: Optimizing Hospital Performance Evaluation in Total Weight Loss Outcomes After Bariatric Surgery: A Retrospective Analysis to Guide Further Improvement in Dutch Hospitals
Source: Obes Surg. 2024 Jul 9;34(8):2820–7. doi: 10.1007/s11695-024-07195-4 (PMC11289147; doi:10.1007/s11695-024-07195-4)
Supplement: Supplementary file 5 — Supplementary file5 (DOCX 160 KB) [file 11695_2024_7195_MOESM5_ESM.docx]

**Appendix -** Statistical code to create a funnel plot around the median for a continuous outcome.

# Use any dataframe of interest containing individual patients and the variables to be analyzed

df_median_funnel = *your_dataframe* %>%

rename(outcome = ‘*your continuous outcome*’) %>% # e.g., %TWL

select(provider, outcome) # provider = e.g., hospital

grand_median = median(df_median_funnel$outcome)

median_per_level <- df_median_funnel %>%

group_by(provider) %>%

summarise(med = median(outcome))

n_per_level <- df_median_funnel %>%

group_by(provider) %>%

summarise(n_patients = n())

median_n_per_level <- left_join(median_per_level, n_per_level, "provider")

N = max(n_per_level$n_patients)

# calculate limits for values in sequence below:

n_patients <- c(seq(2,10,2), seq(12,N+20,10))

n_samples = 10^4

samples = replicate(n_samples, {

df_median_funnel$outcome[unlist(mapply(sample, x = nrow(df_median_funnel), size = n_patients))]})

volume_sequence <- rep(1:length(n_patients), n_patients)

sample_medians <- apply(samples, 2, function(col){

tapply(col, volume_sequence, median)

})

# Create confidence interval

lowerbound <- apply(sample_medians, 1, quantile,0.025)

upperbound <- apply(sample_medians, 1, quantile,0.975)

cidf <- data.frame(n_patients, lowerbound, upperbound)

# calculate limits for specific n per hospital and assign their performance

n_patients_your_df <- median_n_per_level$n_patients

samples_your_df = replicate(n_samples, {

df_median_funnel$outcome[unlist(mapply(sample, x = nrow(df_median_funnel), size = n_patients_your_df))]

})

volume_sequence <- rep(1:length(n_patients_your_df), n_patients_your_df)

sample_medians_your_df <- apply(samples_your_df, 2, function(col){

tapply(col, volume_sequence, median)

})

lowerbound_your_df <- apply(sample_medians_your_df, 1, quantile,0.025)

upperbound_your_df <- apply(sample_medians_your_df, 1, quantile,0.975)

cidf_your_df <- data.frame(n_patients_your_df, lowerbound_your_df, upperbound_your_df)

median_nN_per_level <- left_join(median_n_per_level, cidf_your_df, by = c("n_patients" = "n_patients_your_df"))

median_nN_per_level <- median_nN_per_level %>%

mutate(performance = as.factor(case_when(

med < lowerbound_your_df ~ 1L,

med >= lowerbound_your_df & med <= upperbound_your_df ~ 2L,

med > upperbound_your_df ~ 3L,

TRUE ~ NA_integer_

)),

performance_label = factor(performance,

levels = c(3,2,1),

labels = c("Outperformer", "Average performer", "Underperformer")))

# Create funnel plot

plotmed <- ggplot(data = median_nN_per_level, aes(x = n_patients, y = med))+

geom_point(aes(x = n_patients, y = med, fill = performance_label), shape = 21) +

scale_fill_manual(values = c("green", "grey", "red")) +

geom_line(data = cidf, aes(x = n_patients, y = lowerbound)) +

geom_line(data = cidf, aes(x = n_patients, y = upperbound)) +

geom_hline(aes(yintercept = grand_median))
